# Molecular Modeling Study of Chiral Separation and Recognition Mechanism of β-Adrenergic Antagonists by Capillary Electrophoresis

**DOI:** 10.3390/ijms13010710

**Published:** 2012-01-11

**Authors:** Wuhong Li, Changhai Liu, Guangguo Tan, Xinrong Zhang, Zhenyu Zhu, Yifeng Chai

**Affiliations:** 1 School of Pharmacy, Second Military Medical University, Shanghai 200433, China; E-Mails: nkwuhongli@126.com (W.L.); liuchanghai1976@126.com (C.L.); guangguotan@gmail.com (G.T.); zxr20111963@126.com (X.Z.); zzycyy@yahoo.com.cn (Z.Z.); 2 Shanghai Key Laboratory for Pharmaceutical Metabolite Research, Second Military Medical University, Shanghai 200433, China

**Keywords:** molecular docking, cyclodextrin, β-adrenergic antagonists, capillary electrophoresis, chiral recognition mechanism

## Abstract

Chiral separations of five β-adrenergic antagonists (propranolol, esmolol, atenolol, metoprolol, and bisoprolol) were studied by capillary electrophoresis using six cyclodextrins (CDs) as the chiral selectors. Carboxymethylated-β-cyclodextrin (CM-β-CD) exhibited a higher enantioselectivity power compared to the other tested CDs. The influences of the concentration of CM-β-CD, buffer pH, buffer concentration, temperature, and applied voltage were investigated. The good chiral separation of five β-adrenergic antagonists was achieved using 50 mM Tris buffer at pH 4.0 containing 8 mM CM-β-CD with an applied voltage of 24 kV at 20 °C. In order to understand possible chiral recognition mechanisms of these racemates with CM-β-CD, host-guest binding procedures of CM-β-CD and these racemates were studied using the molecular docking software Autodock. The binding free energy was calculated using the Autodock semi-empirical binding free energy function. The results showed that the phenyl or naphthyl ring inserted in the hydrophobic cavity of CM-β-CD and the side chain was found to point out of the cyclodextrin rim. Hydrogen bonding between CM-β-CD and these racemates played an important role in the process of enantionseparation and a model of the hydrogen bonding interaction positions was constructed. The difference in hydrogen bonding formed with the –OH next to the chiral center of the analytes may help to increase chiral discrimination and gave rise to a bigger separation factor. In addition, the longer side chain in the hydrophobic phenyl ring of the enantiomer was not beneficial for enantioseparation and the chiral selectivity factor was found to correspond to the difference in binding free energy.

## 1. Introduction

β-Adrenergic antagonists are effectively used for the treatment of hypertension, prevention of anginal attacks, suppression of cardiac arrhythmia, prevention of myocardial infarction and possibly, amelioration of congestive heart failure [1]. Most of these drugs are chiral. It is well known that the pharmacodynamic and pharmacokinetic properties of these β-adrenergic antagonist enantiomers differ significantly. For example, β-blocking activity is predominantly related to *S*-propranolol, which is about 100 times more potent than *R*-propranolol [2,3]. Therefore, chiral separations of β-adrenergic antagonists are considered to be an essential issue.

Analytical methods so far used for chiral separations of these β-adrenergic antagonists include thin layer chromatography [4,5], HPLC [6–8], capillary electrochromatography [9,10], and capillary electrophoresis (CE) [11–14]. CE especially has attracted the greater interest for chiral separations. It has a number of advantages in chiral separations such as high separation efficiency, low solvent and selector consumption as well as the ability to readily change the types and concentration of chiral selectors [15]. Numerous chiral selectors (e.g., cyclodextrins (CDs), chiral crown ethers, proteins, chiral surfactants, macrocyclic antibiotics, ligand-exchange complexes, and polysaccharides) are currently available. Among them, CDs are the most widely used due to their excellent chiral recognition abilities. Enantioseparation can be achieved using chiral selectors, which discriminate between the enantiomers by an enantioselective complexation between the enantiomers of the analyte and the chiral selector, giving rise to differences in the electrophoretic mobility of the enantiomers. Chiral recognition mechanism, when CDs are used as chiral selectors, is usually based on inclusion complexation where the analyte fits into the CD cavity. Although some researchers [16–18] have studied chiral recognition mechanisms of β-adrenergic antagonists with CD by NMR, detailed mechanisms underlying the separation remain unknown. The influence of hydrogen bonding in the chiral recognition process was especially difficult to explain due to the limitations of the experimental methods. Fortunately, molecular modeling methods have been recently proposed as powerful tools to obtain information about the emerging interaction of inclusion complexes between CDs and enantiomers and then to elucidate chiral recognition processes [19]. In recent years, various molecular modeling studies such as PM6 semi-empirical methods [20,21], molecular docking [22–24] and molecular dynamics simulations [25–28] have been performed to investigate CD inclusion complexes with the aim of comprehending the mechanism of the complexation and to correlate with the experimental results. The current study applied Autodock molecular docking technique to gain insight into the selector-enantiomer interaction energy and provided useful supporting information for enantiomeric separation.

**Figure 1:**
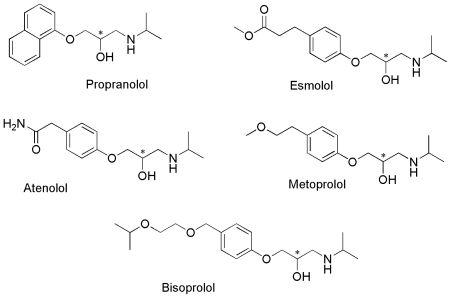
The structures of five β-adrenergic antagonists.

In this study, CM-β-CD was used as the chiral selector (target) and five β-adrenergic antagonists (Figure 1) as the ligands. The influences of chiral selector and its concentration, buffer pH, buffer concentration, temperature and applied voltage on chiral separation of these enantiomers were studied. The course of host-guest inclusion was determined by means of a molecular docking technique and then the binding free energy was calculated using the Autodock semi-empirical binding free energy function. Based on the simulation data of molecular docking and the experimental results of chiral separation by CE, the chiral recognition mechanism of β-adrenergic antagonists with CM-β-CD was studied and the influence of hydrogen bonding in the chiral recognition process was explained.

## 2. Experimental

### 2.1. Apparatus

All CE experiments were performed on an HP^3D^ CE system from Agilent Technologies (Palo Alto, CA, USA) with an online diode-array detector. Instrument control and data acquisition were performed by the HP^3D^ CE ChemStation software [29]. An uncoated fused-silica capillary with a total length of 48.5 cm (effective length 40 cm) × 50 μm i.d. (Yongnian Optical Fiber Plant, Hebei, China) was used as the separation column. Before the first use, the new capillary column was rinsed with methanol for 15 min, followed by 0.1 M hydrochloric acid solution for 15 min, and then activated by flushing with 1 M NaOH for 30 min. Every day the capillary was rinsed with 0.1 M NaOH for 15 min, water for 5 min, and conditioned with background electrolyte (BGE) for 15 min. Between each run, it was treated with 0.1 M NaOH for 4 min, water for 2 min, and running BGE for 4 min. The samples were injected at a pressure of 50 mbar for 3 sec and separated using a constant voltage of 24 kV. The detection wavelength was 214 nm and the column temperature was kept at 20 °C. An ORION Model 828 pH meter with a precision of 0.01 pH units (Thermo Electron, Milford, MA, USA) was used for pH measurement.

### 2.2. Chemicals

Propranolol, esmolol, atenolol, metoprolol, and bisoprolol were purchased from the Chinese Institute of Biological Products Control (Beijing, China). β-CD was obtained from Shanghai San Jie Biochemistry Technology Co., Ltd. (Shanghai, China). Hydroxypropyl-β-CD (HP-β-CD), heptakis-(2,6- di-*O*-methyl)-β-CD (DM-β-CD), heptakis-(2,3,6-tri-*O*-methyl)-β-CD (TM-β-CD) were purchased from Sigma-Aldrich. Sulfated-β-CD (S-β-CD) and carboxymethylated-β-cyclodextrin (CM-β-CD) with purity higher than 98.0% were synthesized by Department of Organic Chemistry, School of Pharmacy, Second Military Medical University (Shanghai, China) and they were identified by IR, NMR, and ESI-MS. Tris was purchased from Shanghai Shisheng Cell Biotechnology Co., Ltd. (Shanghai, China). Phosphoric acid (H_3_PO_4_) was obtained from Shanghai No. 4 Reagent Factory Kunshan Branch (Jiangsu, China) and deionized water was prepared by Milli-Q System (Millipore, Bedford, MA, USA). All other reagents were of analytical grade.

### 2.3. Preparation of BGE Solutions and Analytes

The running BGE consisted of 50 mM Tris and 8 mM CM-β-CD unless otherwise stated. The pH of the buffer was adjusted with phosphoric acid (H_3_PO_4_).

Stock solutions of five drugs were dissolved in 50 mM Tris buffer at a concentration of 10 mM. The samples were prepared by diluting the stock solutions with Tris buffer to a concentration of 0.2 mM before injection.

All solutions were filtered through a membrane filter (0.22 μm) and then stored at 4 °C until analysis.

### 2.4. Molecular Construction and Optimization

Enantiomer construction in its protonated state was carried out using the Sketch Molecule module of SYBYL7.0 software package [30]. The molecular mechanics Powell method [31] was applied for structure optimization and charges were considered by using the Gasteiger-Huckel charge calculation. MOPAC 6.0 [32] was used to adjust the spatial unreasonable bond distances and bond angles of all enantiomers [33,34].

The three-dimensional structure of CM-β-CD was constructed taking as reference β-CD coordinates extracted from the β-CD_α-hemolysin complex crystal structure (PDB entry code: 3M3R [35]). They were then optimized using the molecular mechanics Powell method and charges were considered by using the Gasteiger-Huckel charge calculation in SYBYL7.0 software package. The structure of CM-β-CD was derived from the three-dimensional structure of β-CD. All molecular mechanics calculations and quantum chemistry calculations were carried out on an Origin 300 Server.

### 2.5. Docking and Mechanic Calculations

Autodock 3.0 [36] software was applied for molecular docking and the software had been validated for simulating the interactions of two molecules [37]. The optimized drug enantiomers were inserted into the hydrophobic cavity of CM-β-CD. The Lamarckian genetic algorithm in Autodock 3.0 was applied to search the conformational and orientational space and the number of iterations for the local search was 300 using the Solis and Wets algorithm. The most stable docking conformations were selected on the basis of docking energies and the first pose of the most populated cluster. The binding free energy Δ*G* was calculated from the most stable docking conformations using Autodock semi-empirical binding free energy function [38]:

(1) $$\begin{array}{l} \Delta G=\Delta G_{vdw}\sum_{i,j}\left(\frac{A_{i,j}}{r_{ij}^{12}}-\frac{B_{i,j}}{r_{ij}^{6}}\right)+\Delta G_{hbond}\sum_{i,j}E(t)\left(\frac{C_{i,j}}{r_{ij}^{12}}-\frac{D_{i,j}}{r_{ij}^{10}}\right)+\Delta G_{elec}\sum_{i,j}\frac{q_{i}q_{j}}{ɛ(r_{ij})r_{ij}}\\ +\Delta G_{tor}N_{tor}+\Delta G_{sol}\sum_{i,j}{\left(S_{i}V_{j}+S_{j}V_{i}\right)}^{-r_{ij}^{2}/2\sigma^{2}} \end{array}$$

where the first three terms are the typical molecular mechanics terms for dispersion/repulsion, hydrogen bonding, and electrostatics, respectively; Δ*G*_tor_ term models the restriction of internal rotors and global rotation and translation; and Δ*G*_sol_ term models desolvation upon binding and the hydrophobic effect (solvent entropy changes at solute-solvent interfaces). The magnitude of the binding free energy change indicates the tendency towards complexation. The more negative the binding free energy change is, the more thermodynamically favorable is the inclusion complex.

### 2.6. Chromatographic Thermodynamics Calculations

The equilibrium constant, *K*_i_ can be obtained from the binding free energy using Equation (2).

(2) $$\Delta G=-RT\;\text{ln }K_{i}$$

where *R* is the gas constant, and *T* is the absolute temperature. Additionally, the separation factor, which is also named selectivity factor, is approximately calculated by the time of the first and second migrating enantiomer determined by CE using the Equation (3).

(3) $$\alpha =t_{R}/t_{S}$$

Moreover, separation factor can also be defined as Equation (4) [39,40]:

(4) $$\alpha =K_{R}/K_{S}$$

Where *K*_R_ is the equilibrium constant of *R-*enantiomer and *K*_s_ is the equilibrium constant of *S-*enantiomer, assumed to be *K*_R_ > *K*_S_. Therefore, the absolute value of the difference of the binding free energy can be calculated using Equation (5).

(5) ∣ΔΔG∣=RT ln α

## 3. Results and Discussion

### 3.1. Optimization of Chiral Separations

#### 3.1.1. Effect of the Type of CD

The type of CD plays an important role in enantiomeric separation by cyclodextrin-capillary zone electrophoresis. The discrimination power of these six CDs was investigated by means of a preliminary screening test. Analysis tests were performed with a 50 mM Tris (pH 3.0) buffer individually containing CM-β-CD 8 mM, S-β-CD 8 mM, HP-β-CD 30 mM, DM-β-CD 30 mM, TM-β-CD 30 mM, and β-CD 16 mM. Large differences in chiral resolution were obtained for the analytes using these different CDs. Separation factors obtained from the chiral separations of five drug racemates with the six CDs are presented in Table 1. Only enantiomeric separations of propranolol were observed on using β-CD as the chiral selector. Partial enantiomeric separations of several drug racemes were achieved with HP-β-CD, DM-β-CD, TM-β-CD and S-β-CD. However, all the drugs could be separated using CM-β-CD as chiral selector. CM-β-CD, compared to the other CDs, had the better chiral recognition abilities for these enantiomers perhaps due to the carboxymethylated group. Hence, CM-β-CD was chosen for the optimization of the separation.

#### 3.1.2. Effect of Buffer pH

**Figure 2:**
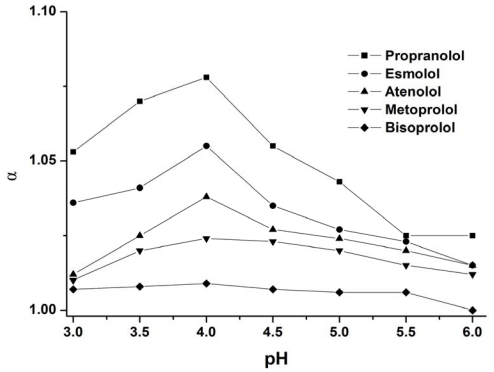
Effect of buffer pH on the selectivity factor of five drug enantiomers. Conditions: uncoated fused-silica capillary, 48.5 cm (effective length 40 cm) × 50 μm i.d.; BGE, 50 mM Tris buffer containing 8 mM CM-β-CD; UV detection at 214 nm; 50 mbar for 3 sec; temperature, 20 °C; applied voltage, 24 kV.

The pH of the buffer is another important parameter affecting chiral separation. Effective charge and mobility of the analyte and CM-β-CD directly depends on the pH. Experiments were performed using buffer at different pH values in the range of 3.0–6.0 obtained by addition of increasing H_3_PO_4_ amounts to Tris solution. The influence of the pH of the buffer on the enantioseparation of five drugs was shown in Figure 2. The variation of the pH had the same effect on the chiral separation factor for the five drugs. The chiral separation factor reached a maximum value at pH 4.0. Therefore, the buffer of pH 4.0 was selected for the optimal enantioseparation method.

#### 3.1.3. Effect of the CD Concentration

**Figure 3:**
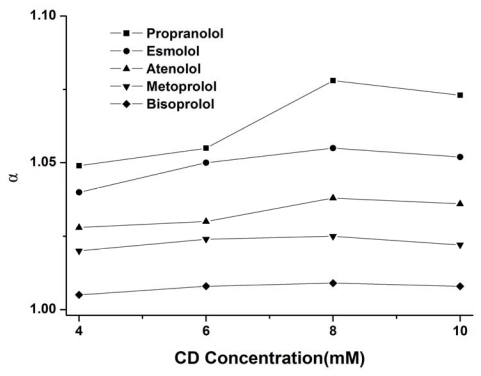
Effect of the carboxymethylated-β-cyclodextrin (CM-β-CD) concentration on the selectivity factor of five drug enantiomers. Conditions: uncoated fused-silica capillary, 48.5 cm (effective length 40 cm) × 50 μm i.d.; BGE, 50 mM Tris buffer (pH = 4.0) containing CM-β-CD; UV detection at 214 nm; 50 mbar for 3 sec; temperature, 20 °C; applied voltage, 24 kV.

The CD concentration is also an essential parameter for the optimization of chiral separations because the concentration of the chiral selector affects directly the affinity of the enantiomers for the selector [41]. The influence of the concentration of CM-β-CD in the separation buffer on the chiral separation of these drugs was investigated in the range from 4 to 10 mM using a 50 mM Tris buffer (pH 4.0). As shown in Figure 3, the variation of the CD concentration had also the same effect on the chiral resolution for the five drugs. The chiral separation factor reached a maximum value at a concentration of 8 mM. A further increase in concentration could result in a slow decrease in the separation factor. This trend further confirmed the theoretical model developed by Wren and Rowe [42] concerning the existence of a maximum selectivity at a certain concentration of chiral selector. Therefore, the 8 mM concentration of CM-β-CD was chosen for further optimization of the separation.

#### 3.1.4. Effect of the Buffer Concentration, Voltage, and Temperature

The effect of the concentration of the buffer on the separation was investigated using Tris buffer (pH 4.0) in the concentration range 30–60 mM for the separation. Increase of the buffer concentration lowers the electroosmotic flow (EOF) and increases the current and the temperature. With the increase of the buffer concentration, the separation factor of five drugs changed slightly. Hence, 50 mM Tris buffer was selected for the optimal enantioseparation method. The effect of applied voltage in the range of 20–30 kV was studied. Application of higher voltage (26–30 kV) resulted in an obvious decrease in the separation factor, due to the increased Joule heating. However, use of lower voltage (20–22 kV) resulted in longer migration times. So the optimal voltage for analysis was chosen as 24 kV. The effect of the temperature in the range of 15–25 °C on the separation factor and migration time of five drugs was investigated. The increase in the temperature leads to a reduction of migration times by decreasing the buffer viscosity. However, higher temperature could enhance the band broadening and thus decrease the peak efficiency and the resolution. Therefore, a temperature of 20 °C was chosen for the final method.

#### 3.1.5. Optimum Conditions for Enantioseparation

**Figure 4:**
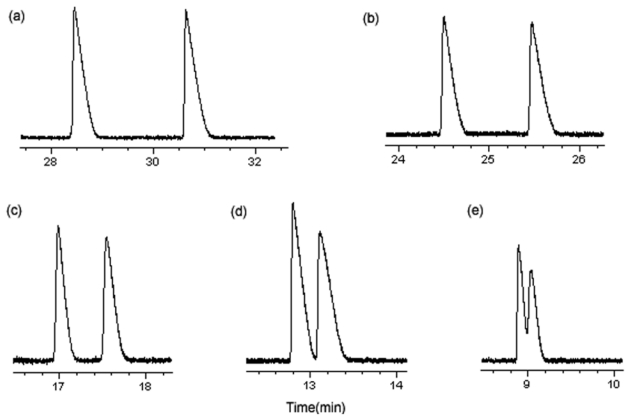
Electropherograms of five β-adrenergic antagonists under the optimal conditions: (**a**) propranolol; (**b**) esmolol; (**c**) atenolol; (**d**) metoprolol; and (**e**) bisoprolol.

After optimization of the various factors, the final method was as follows: uncoated fused-silica capillary, 48.5 cm (effective length 40 cm) × 50 μm i.d.; BGE, 50 mM Tris buffer, pH 4.0, with 8 mM CM-β-CD; UV detection at 214 nm; injection, 50 mbar for 3 sec of 0.2 mM solution; temperature, 20 °C; applied voltage, 24 kV. The separation factors obtained for five β-adrenergic antagonists in these optimal conditions were listed in Table 2 and the electropherograms were presented in Figure 4.

### 3.2. Molecular Docking and Chiral Recognition Mechanism

**Figure 5:**
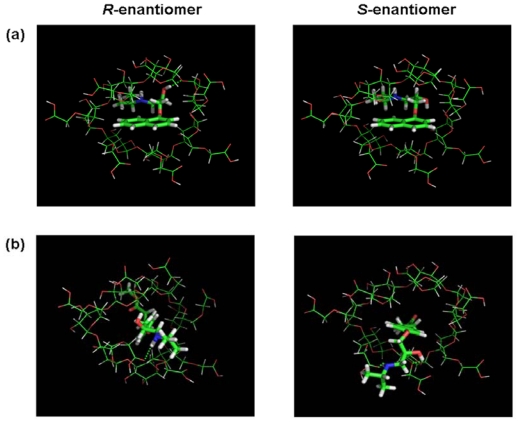

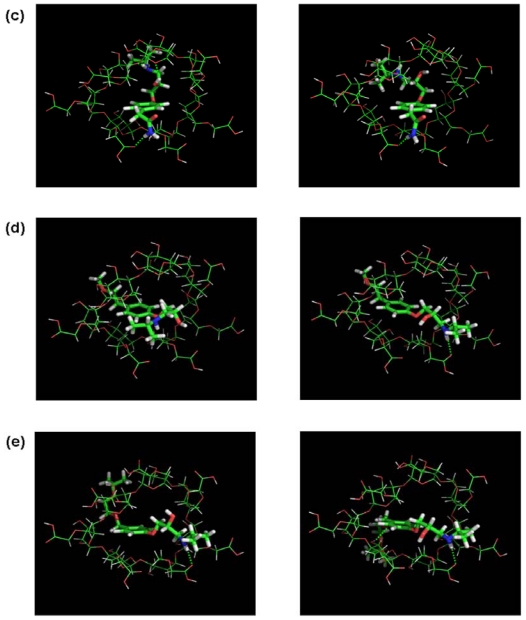
The molecular docking configuration between five β-adrenergic antagonist enantiomers and CM-β-CD: (**a**) propranolol; (**b**) esmolol; (**c**) atenolol; (**d**) metoprolol and (**e**) bisoprolol. The left is *R*-enantiomer and the right is *S*-enantiomer. The hydrogen bonding is indicated with the green dashed line.

**Figure 6:**
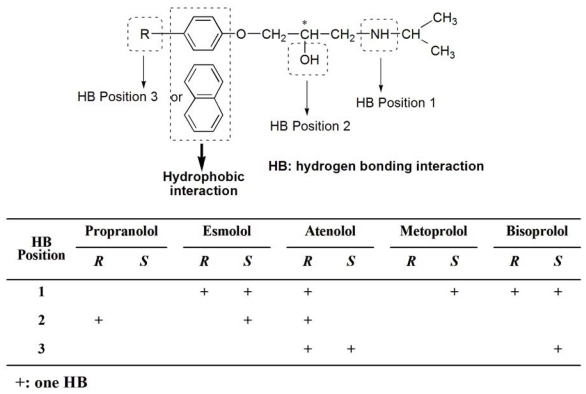
Model and summary table of hydrogen bonding interaction of carboxymethylated-β-cyclodextrin(CM-β-CD) with β-adrenergic antagonist enantiomers.

Molecular modeling is frequently used to rationalize experimental findings concerning chiral recognition by CDs due to the limitations of the experimental methods. The methods are valuable tools for obtaining information on the geometry and the interaction energy of the inclusion compounds. To understand possible chiral recognition mechanisms of these racemates with CM-β-CD, host-guest binding procedures of CM-β-CD and these racemates were studied using the molecular docking software Autodock 3.0. The molecular docking configuration of five drugs and CM-β-CD was shown in Figure 5. A hydrophobic naphthyl or phenyl ring of these racemates inserted in the hydrophobic cavity of CM-β-CD and the side chain were found to point out of the cyclodextrin rim. The molecular docking configuration of metoprolol and CM-β-CD had been confirmed by NMR studies [16]. Although the electrostatic interaction between the positively charged nitrogens of the side chain and the negative charges of CM-β-CD was probably the main chiral recognition interaction [43], the intermolecular hydrogen bonding in the chiral recognition process could not be underestimated because it could be formed between hydrogen donors or acceptors in the side chain and on the cyclodextrin rim. Hydrogen bonding interactions of these racemates with CM-β-CD were summarized from the molecular docking studies in Figure 6. Based on the findings from the summary of hydrogen bonding and the result of molecular docking studies, a model was generated (see Figure 6).

In this model there were three hydrogen bonding interaction position (–NH, –OH and the other groups in the side chain) and a hydrophobic interaction position. The hydrophobic interaction between the phenyl or naphthyl group and the cavity of cyclodextrin may help in forming stable host-guest complexes and the difference in hydrogen bonding may increase chiral discrimination besides electrostatic interaction between the positively charged nitrogens of the side chain and the negative charges of CM-β-CD. Particularly, the difference in hydrogen bonding formed with the –OH next to the chiral center may help to increase chiral discrimination and give rise to the higher separation factor such as propranolol, esmolol, and atenolol. Propranolol was able to be separated using all the CDs. Interestingly, propranolol had the highest separation factor of the five drugs regardless of the type of cyclodextrin as listed in Table 1. It might be attributable to the strongly hydrophobic naphthyl group of propranolol. The strong plane of naphthalene helped in easily forming a stable inclusion complex. Also, naphthalene had a larger structure than the phenyl ring, which gave rise to lower spatial degrees of freedom in the hydrophobic cavity of CM-β-CD as shown in Figure 5. In addition, the separation factors of esmolol, atenolol, metoprolol, and bisoprolol, with a hydrophobic phenyl ring in their structures, gradually decreased. Considering the structure of these four drugs, the substitutions on the phenyl ring had longer side chains, from esmolol to bisoprolol, which may be unfavorable to host-guest binding procedures due to less flexibility and steric hindrance. It is suggested that the longer side chain in the hydrophobic phenyl ring of the enantiomer is not good for enantioseparation. All these differences in the drug enantiomers probably gives rise to the difference in binding energy in the enantiorecognition process and then to the chiral discrimination.

The difference in energies of the inclusion complexes between the enantiomers and CD is probably a measure of chiral discrimination, which results in the separation of the enantiomers and the different separation factors as observed in the experimental studies. Therefore, the binding free energy Δ*G* in the course of inclusion between each enantiomer and CM-β-CD was calculated using Equation (1) and the results were listed in Table 2. All CM-β-CD-analyte inclusion complexes had binding free energies in the range of −3.410 to −5.984 kcal/mol. The negative values for Δ*G* for all the complexes indicated the spontaneity of the binding of the guest molecule to the host. The calculated and experimental absolute values of the difference of the binding free energy |ΔΔ*G|* were also calculated using the stated equation and the results were also listed in Table 2. It showed that the calculated |ΔΔ*G|* magnitudes were in the order propranolol > esmolol > atenolol > metoprolol > bisoprolol, for the different structures of the side chains. The experimental |ΔΔ*G|* and chiral selectivity factor magnitudes had also the same order, which indicated that the chiral selectivity factor corresponded with the difference in binding free energy. However, the values between the calculated and experimental |ΔΔ*G|* had a significant difference in the range of 0.135 to 0.414 and 0.022 to 0.183 kcal/mol, respectively. A number of assumptions are usually encountered in modeling chiral separations of chromatographic techniques. Such factors as buffer effect, solvation effect as well as entropy difference were usually not considered. This could lead to the significant difference between the calculated and experimental |ΔΔ*G|*. In addition, the values of experimental |ΔΔ*G|* were also approximately calculated using Equation (5). However, the combination of molecular docking and CE experiment results could help us predict the enantioseparation of the other structurally related analytes under given experimental conditions.

## 4. Conclusions

Chiral separations of five β-adrenergic antagonists were achieved by capillary electrophoresis using CM-β-CD as the chiral selectors. The good chiral separations of five β-adrenergic antagonists were achieved using 50 mM Tris buffer at pH 4.0 containing 8 mM CM-β-CD with an applied voltage of 24 kV at 20 °C on investigating the influences of several important analytical conditions. The host-guest binding procedures of CM-β-CD and these drug racemates were studied and their binding free energies were calculated using the molecular docking software Autodock 3.0. According to the simulation data of molecular docking combined with the experimental results of chiral separation by CE, the results achieved were explained as follows: (i) Hydrogen bondings between CM-β-CD and these racemates play an important role in the process of enantionseparation; (ii) A longer side chain in the hydrophobic phenyl ring of enantiomer was not good for enantioseparation; (iii) The chiral selectivity factor corresponded to the difference in binding free energy. It should be noted that a different enantio-recognition mechanism may be achieved using the other CDs as the chiral selector. These findings are only related to CM-β-CD as the chiral selector. Although it cannot completely elucidate chiral recognition mechanisms based alone on CE separation data and molecular modeling results, the molecular modeling technique, however, provides us with a good perspective of enantio-separation and serves as a useful method for studying chiral recognition mechanisms.

## Figures and Tables

**Table 1:** Influence of the cyclodextrin (CD) type on the enantioseparation of five β-adrenergic antagonists.

Enantiomers	Selectivity Factors (α)					

	β-CD	HP-β-CD	DM-β-CD	TM-β-CD	S-β-CD	CM-β-CD
Propranolol	1.009	1.016	1.010	1.015	1.019	1.053
Esmolol	1.000	1.012	1.007	1.010	1.006	1.036
Atenolol	1.000	1.006	1.000	1.000	1.007	1.012
Metoprolol	1.000	1.000	1.000	1.000	1.000	1.010
Bisoprolol	1.000	1.000	1.000	1.000	1.000	1.007

HP: hydroxypropyl; DM: heptakis-(2,6-di-*O*-methyl); TM: heptakis-(2,3,6-tri-*O*-methyl); S: sulfated; CM: carboxymethylated.

**Table 2:** Binding free energy of carboxymethylated-β-cyclodextrin (CM-β-CD) with five drug enantiomers and selectivity factors.

Enantiomers	Binding Free Energy (ΔG) (kcal/mol)		Difference of Binding Free Energy (|ΔΔG|) (kcal/mol)		Selectivity Factors (α) **

	*R*-ΔG*	*S*-ΔG*	Calculated	Experimental	
Propranolol	−4.340	−3.926	0.414	0.183	1.078
Esmolol	−5.113	−5.469	0.356	0.131	1.055
Atenolol	−5.984	−5.679	0.305	0.091	1.038
Metoprolol	−3.410	−3.622	0.212	0.057	1.024
Bisoprolol	−4.612	−4.747	0.135	0.022	1.009

* Average energy of the best cluster (the lowest docked energy);

** Conditions are the same as optimum conditions.
